# Patient-derived tumor immune microenvironments in patient-derived xenografts of lung cancer

**DOI:** 10.1186/s12967-018-1704-3

**Published:** 2018-11-26

**Authors:** Xingxiang Pu, Ran Zhang, Li Wang, Yungchang Chen, Yi Xu, Apar Pataer, Ismail M. Meraz, Xiaoshan Zhang, Shuhong Wu, Lin Wu, Dan Su, Weimin Mao, John V. Heymach, Jack A. Roth, Stephen G. Swisher, Bingliang Fang

**Affiliations:** 1 Department of Thoracic Medical Oncology, Hunan Cancer Hospital/The Affiliated Cancer Hospital of Xiangya School of Medicine, Central South University, 283 Tongzipo Road, Yuelu District, Changsha, 410013 Hunan China; 2 Department of Thoracic and Cardiovascular Surgery, The University of Texas MD Anderson Cancer Center, Houston, TX 77030 USA; 3 Department of Pathology, Zhejiang Cancer Hospital, 38 Guanji Road, Banshan Bridge, Hangzhou, 310022 Zhejiang China; 4 Department of Thoracic Surgery, Zhejiang Cancer Hospital, 38 Guanji Road, Banshan Bridge, Hangzhou, 310022 Zhejiang China; 5 Department of Thoracic/Head and Neck Medical Oncology, The University of Texas MD Anderson Cancer Center, Houston, TX 77030 USA

**Keywords:** Lung cancer, Patient-derived xenografts (PDX), Tumor models, Tumor-infiltrating lymphocytes, Immunotherapy, Tumor microenvironment

## Abstract

**Background:**

Because patient-derived xenografts (PDXs) are grown in immunodeficient mouse strains, PDXs are regarded as lacking an immune microenvironment. However, whether patients’ immune cells co-exist in PDXs remains uncharacterized.

**Methods:**

We cultured small pieces of lung PDX tissue in media containing human interleukin-2 and characterized the proliferated lymphocytes by flow cytometric assays with antibodies specific for human immune cell surface markers. Presence of immune cells in PDXs was also determined by immunohistochemical staining.

**Results:**

Human tumor-infiltrating lymphocytes (TILs) were cultured from nine of 25 PDX samples (36%). The mean time of PDX growth in immunodeficient mice before obtaining TILs in culture was 113 days (range 63–292 days). The TILs detected in PDXs were predominantly human CD8^+^ T cells, CD4^+^ T cells, or CD19^+^ B cells, depending on cases. DNA fingerprint analysis showed that the TILs originated from the same patients as the PDXs. Further analysis of two PDX-derived CD8^+^ T cells showed that they were PD-1^−^, CD45RO^+^, and either CD62L^+^ or CD62L^−^, suggesting they were likely memory T cells. Immunohistochemical staining showed that human T cells (CD8^+^ or CD4^+^), B cells (CD19^+^), and macrophages (CD68^+^) were present in stroma or intraepithelial cancer structures and that human PD-L1 was expressed in stromal cells. Moreover, the patient-derived immune cells in PDX can be passaged to the F2 generation and may migrate to spleens of PDX-bearing mice.

**Conclusions:**

Patient-derived immune cells co-exist in early passages of PDXs in some lung cancer PDX models. The CD8^+^ cells from PDXs were likely memory T cells. These results suggest that PDXs can be used for evaluating the functionality of immune components in tumor microenvironments.

**Electronic supplementary material:**

The online version of this article (10.1186/s12967-018-1704-3) contains supplementary material, which is available to authorized users.

## Background

Recent progress in immunotherapy with immune checkpoint inhibitors has offered new options for the treatment of lung cancer, a deadly disease that each year causes approximately 155,870 deaths in the United States and 1.6 million deaths worldwide [1, 2]. The anti-PD-1 antibodies nivolumab [3, 4] and pembrolizumab [5, 6] and the anti-PD-L1 antibodies atezolizumab [7] and durvalumab [8] have recently been approved for treatment of non-small cell lung cancer (NSCLC) by the US Food and Drug Administration. Clinical studies have shown that immune checkpoint blockade therapy for patients with advanced NSCLC improved survival rates, prolonged duration of response, and reduced treatment-related adverse effects [9]. Nevertheless, despite the results of clinical trials with PD-1 [4, 5, 10, 11] and PD-L1 [7, 12, 13] inhibitors have been promising, the overall objective response rates in NSCLC patients who received such therapy were about 20%, and most patients had primary resistance [3–5, 7, 10–12]. Strategies to overcome primary resistance to immune checkpoint inhibitors, either through combination therapy or through discovery of new targets to modulate anticancer immunity, are urgently needed so that more lung cancer patients can benefit from immunotherapy. However, preclinical investigation of therapeutic agents that recognize only the human version of immune checkpoint molecules is drastically limited by a lack of proper preclinical models due to interspecies differences in the specificity of checkpoint molecules, growth factors, and cytokines. Thus, mouse models with an in vivo “humanized” environment will be desirable for preclinical evaluation of strategies aimed at improving efficacies of immune-modulating drugs.

Patient-derived xenograft (PDX) models have been shown to recapitulate histologic features, gene expression patterns, and genomic alterations in human primary tumors [14–16] and have emerged as robust preclinical models for drug development, molecular characterization of cancers, and strategic development of precision therapy [17–21]. Our recent study on molecular characterization of lung cancer PDXs found that 93% of mutations found in primary tumors were also found in PDXs, suggesting that PDXs have the ability to recapitulate the mutations in the primary tumors [22]. However, because PDXs are grown in immunodeficient mouse strains, they are regarded as inappropriate for preclinical evaluation of anticancer immunotherapy because of a lack of host immune components. Nevertheless, studies by others have shown that a subgroup of lung cancer patients has intensive lymphocyte infiltration in the tumor stroma and/or tumor nest [23–26]. For example, a study with more than 1500 patients with resectable NSCLC found that about 10% of these patients had intense lymphocytic infiltration in their tumors, and this subset of patients had better overall survival outcomes than did patients with nonintense tumor lymphocytic infiltration [25]. Evidence has shown that co-transplantation of human immune cells within metastatic melanomas into the subcutis of NOD-SCID IL-2Rγ^null^ (NSG) mice for generating PDXs may lead to graft-versus-host disease, reactive lymphoid infiltrates effacing xenografted tumors, and post-transplant B cell lymphomas associated with Epstein–Barr virus reactivation [27]. It is also reported that intratumoral sustained release of recombinant human IL-12 in human lung tumor tissue implanted into NSG mice resulted in prolonged existence of effector memory T cells and CD138^+^ plasma cells within the tumor xenograft (for up to 9 weeks) [28]. The tumor-associated T cells were also found to migrate from the xenograft to the spleen, lung, and liver of the xenograft-bearing mice, and significant levels of human interferon-γ and immunoglobulin were detected in sera from these mice [28], suggesting that human immune cells co-implanted with tumor tissues could survive and expand in immunodeficient mice. However, it remains undetermined whether patient-derived tumor-infiltrating lymphocytes (TILs) can co-exist within PDXs grown in NSG mice and how long these cells can persist.

To test whether patient-derived immune components co-exist in PDXs, we cultured PDX tumor tissues for TILs in vitro by adding human interleukin-2 (IL-2) to the medium. Our results showed that patient-derived TILs were successfully cultured from PDX samples. The mean time of PDX growth in NSG mice before harvesting to obtain TILs in vitro was about 113 days (range 63–292 days). Moreover, patient-derived TILs can be passed together with PDXs in early passages.

## Materials and methods

### Human lung tissue specimens

Fresh lung cancer samples were collected in 2015 and 2017 from surgically resected specimens under research protocols approved by the Institutional Review Board at The University of Texas MD Anderson Cancer Center. All clinical samples were collected with informed consent from the patients.

### Generation of patient-derived xenografts

PDXs were established from surgically resected specimens or from pleural fluid as we previously reported [22], except that non-obese diabetic/severe combined immunodeficiency (NOD-SCID) mice with null mutations of the gene encoding for the IL-2 receptor-γ (NSG) were used instead of regular NOD-SCID mice for generation and passaging of PDXs. For generating PDXs from fresh surgical specimens, tumor tissue was cut to about 2 mm^3^ in size and implanted into the flank subcutaneous space of mice. For generating PDXs from pleural fluid, the fluid samples were centrifuged to collect cell pellets. Red blood cells in the samples were lysed with sterile buffer (150 mM NH_4_Cl, 20 mM Tris–Cl, pH 7.4), and the pellets were washed twice with phosphate-buffered saline. About 2 × 10^7^ cells were inoculated into the rear flank subcutaneous area of NSG mice. The mice were monitored for up to 12 months for tumor growth. The NSG mice were obtained from Jackson Laboratory (Bar Harbor, ME) or from our institutional Research Animal Support Facility. All animal experiments were performed in accordance with the *Guidelines for the Care and Use of Laboratory Animals* (NIH publication number 85-23) and the institutional guidelines of MD Anderson Cancer Center and were approved by our Institutional Animal Care and Use Committee.

### Culturing TILs from PDX

The mice inoculated with patient tumor samples were monitored for up to 12 months for tumor growth. The tumors were harvested for cryopreservation, passage, or cell culture when they reached 1.5 cm in diameter. For culturing TILs, small pieces (about 1–2 mm^3^ in size) of fresh tumor tissues were placed into a petri dish with 3 to 4 mL of Roswell Park Memorial Institute (RPMI) medium and minced with a scalpel. The minced samples were centrifuged briefly. After they were washed with medium, the pellets were suspended in 2 to 5 mL of RPMI 1640 medium with 10% fetal bovine serum, 100 µg/mL penicillin–streptomycin (all from Invitrogen, Carlsbad, CA), and 2000 to 3000 units/mL of human IL-2 (SYD Labs, Natick, MA). After the 1st week in culture, half of the volume of medium from each well was replaced with fresh medium three times per week. Cells were grown or maintained at a cell concentration of 0.5 − 2 × 10^6^ cells/mL for up to 4 weeks. Cell cultures were maintained at 37 °C in an incubator with 95% humidity and 5% CO_2_.

### Flow cytometric analysis

Cultured TILs were characterized by flow cytometric assays for cell surface biomarkers. For tissue samples (tumor or spleen), small pieces of tissue fragments were digested with collagenase (1 mg/mL) and DNase I (50 ng/mL) in serum-free RPMI 1640 medium overnight at 4 °C. The digested suspension was passed through sterile 40-µm cell strainer and then washed twice with PBS. The cell suspension was stained with Live/Dead fixable violet (Invitrogen) and corresponding monoclonal antibodies. The following panel of mouse anti-human monoclonal antibodies was used in the flow cytometric assays: anti-human CD45-APC/Cy7 (BioLegend, San Diego, CA; clone 2D1), anti-human CD3-PerCP/Cy5.5 (BioLegend, HIT3a), anti-human CD4-PE (BD Biosciences, San Jose, CA; RPA-T4), anti-human CD8-FITC (BD Biosciences, HIT8a), anti-human CD45RO-FITC (BioLegend, 304204, UCHL1), anti-human 45RA (BioLegend, HI100), anti-human CD62L-APC (BioLegend, DREG-56), anti-human CD19-APC (BioLegend, HIB19), anti-human CD14-FITC (BioLegend, 63D3), and anti-human CD56-PE (BioLegend, HCD56). Rat anti-mouse CD45-Alexa Fluor 700 (BD Biosciences, 300-F11) was used as control for mouse white blood cells. The flow cytometric assay data were acquired using a BD LSRFortessa analytical flow cytometer. Unstained and single fluorochrome-stained cells were used as controls to provide accurate compensation and data analysis. Cells were counted per sample, and the data were analyzed with FlowJo software (version 10).

### DNA fingerprinting

DNA isolated from primary tumor samples, PDXs, and TILs cultured from PDXs were analyzed for provenance by DNA fingerprint assay. This assay was performed at our institutional Characterized Cell Line Core using the PowerPlex 16 HS System (Promega). The short tandem repeat profiles were compared with those of case-matched specimens obtained from the patients and/or with 2455 known profiles in online databases (American Type Culture Collection [ATCC], German Collection of Microorganisms and Cell Culture [DSMZ], Japanese Collection of Research Bioresources [JCRB], and RIKEN Cell Bank) and 2556 known profiles in the MD Anderson Characterized Cell Line Core database.

### Immunohistochemical analysis of PDX

Immunohistochemical staining for formalin-fixed paraffin-embedded tumor tissue slides was performed at the Research Histology, Pathology and Imaging Core of MD Anderson. The sectioning slides were stained with mouse monoclonal antibodies specific for human CD4 (Invitrogen, clone 4B12), CD8 (eBioscience, clone C8/144B), CD19 (Bio-Rad, #MCA2454), CD68 (Dako, clone PG-M1), and PD-L1 (Cell Signaling, clone E1L3N) using the standard operation protocols performed in the Core facility. Mouse immunoglobulin G was used as negative control. Human lymph node samples were used as positive control for anti-human CD4 and CD8 antibodies.

## Results

### Patient-derived xenografts from lung cancer specimens

We investigated in vitro TIL culture from 25 PDXs generated and/or passaged in NSG mice. The demographic information and clinical diagnoses for patients from whom the PDXs were generated are summarized in Additional file 1: Table S1. Patients were 39 to 85 years old when the samples were acquired, with a mean age of 67.8 years. Most patients were former or current smokers. Seventeen patients were women and eight were men; 21 were Caucasian and four were of other ethnicity. Fifteen patients had adenocarcinomas, six had squamous cell carcinomas, and one each had adenosquamous carcinoma, giant/spindle cell cancer, neuroendocrine cancer, and pleomorphic cancer. Most tumors were poorly or moderately differentiated. The tumor sizes varied from 0.72 to 420 cm^3^, with a mean of 93.62 cm^3^. Four patients underwent chemotherapy prior to sample acquisition. The overall engraftment rate of lung cancer PDXs in NSG mice was about 45%, higher than what we observed previously in NOD-SCID mice [22]. Thirteen PDXs were in the first passage in mice (F1), and 12 PDXs were in the second passage (F2) when they were acquired for TIL culture. The total time of PDXs growing in mice ranged from 64 to 514 days, with a mean of 172 days.

### TIL culture from PDXs

Fresh tumor tissues from 25 PDXs were tested for TIL culture with supplemental human IL-2 in the culture medium. TIL was obtained from nine of the 25 PDXs (36%) within about 2 weeks of in vitro culture. Eight of the nine PDXs were derived from adenocarcinoma and one from squamous cell cancer. Seven of these nine were from the first passage and two from the second passage (Table 1). The mean in vivo time of PDXs before harvesting to obtain TILs in culture was 113 days (range 63–292 days). Three PDXs, all from the first passage, were predominantly B cells (based on cell surface biomarker analysis), whereas the remaining six PDXs were predominantly CD8^+^ or CD4^+^ T lymphocytes. Three of four patients who had undergone chemotherapy prior to sample acquisition had TILs cultured from their PDXs (75% vs 36% of overall cases). These three patients had their PDXs derived from surgically resected tissues, while the remaining patient who had received chemotherapy prior to sample acquisition (but did not have TIL in PDX culture) had the PDX derived from pleural fluid. However, due to the small sample sizes, it is not clear whether prior chemotherapy would increase the chance of obtaining patient-derived TILs in PDXs.

**Table 1:** PDXs from which TILS were obtained by culture

Case ID	Passages	In vivo days	In vitro days	CD8^+^ (%)	CD4^+^ (%)	CD19^+^ (%)
1	F1	292	14	99.1	0.5	0.9
4	F1	90	10	72.1	1.6	0
5	F1	128	13	50.3	4.6	0
13	F1	98	10	76.1	20	0.7
2	F1	133	10	1.9	0	95.8
11	F1	63	10	12.3	0.2	87.1
10	F1	69	7	8.7	26.9	77.6
19	F2	84	22	99.2	0	0
16	F2	72	14	23.7	70.2	0

### Cell subtypes of TIL from PDXs

**Fig. 1:**
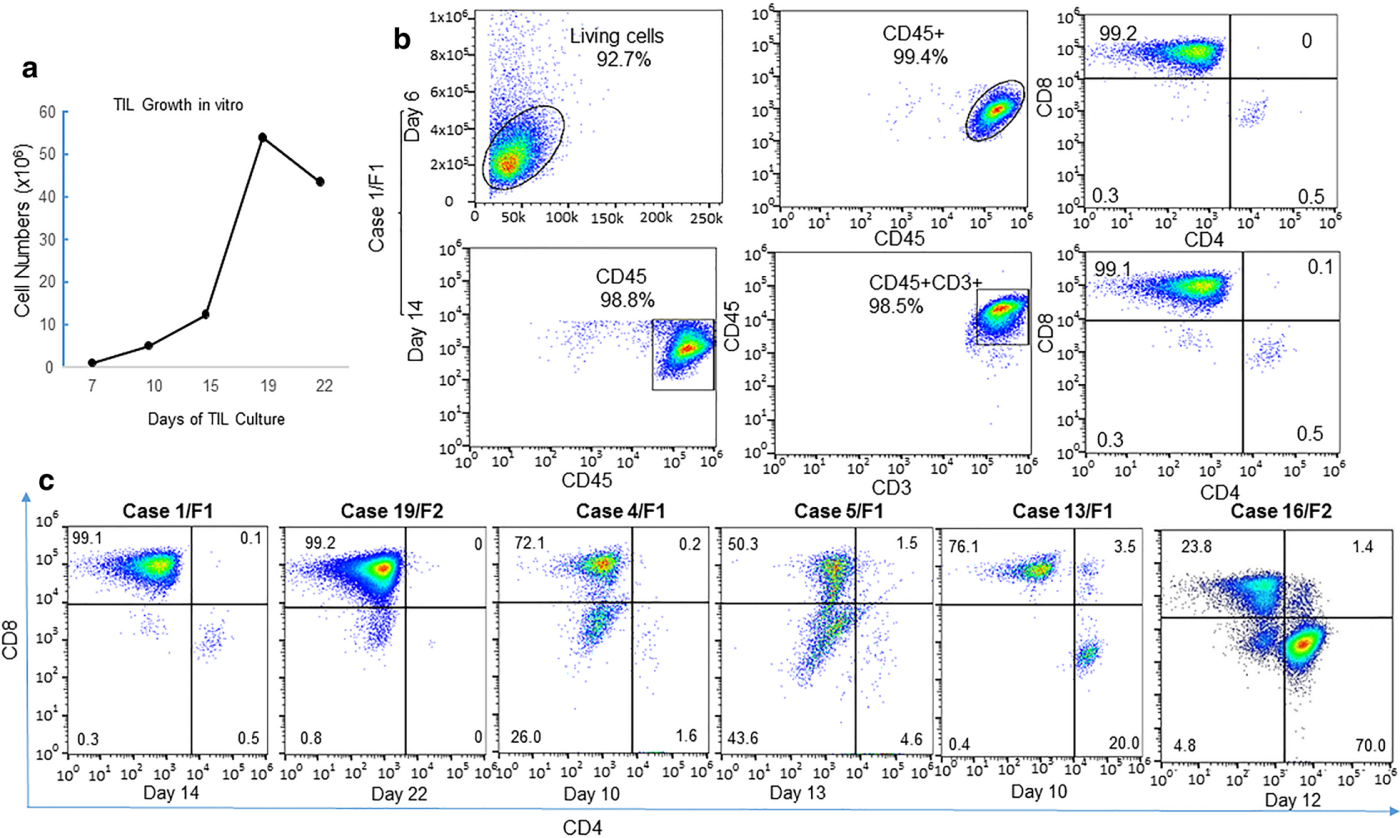
TIL culture and characterization from PDX. **a** An example of TIL growth in vitro in medium containing IL-2 (case 19). **b** Flow cytometric analysis of TILs cultured from case 1 at day 6 and day 14 after culture. **c** Flow cytometric profiles of TILs from six PDXs. The numbers in each panel indicate the percentages of cells in each fraction. All antibodies used were mouse anti-human monoclonal antibodies

Growth of TIL from PDXs was usually observed under the microscope at about 1 week after in vitro culture. Most TILs grew continually in vitro for up to 4 weeks. An example of in vitro growth of PDX-derived TIL is shown Fig. 1a. To determine cell subtypes of TILs derived from the PDXs, we performed fluorescence-activated cell sorting (FACS) analysis for cell surface markers using commercially available antibodies as described in “Materials and methods”. On the basis of cell growth status, FACS was performed at various time points during the 2nd or 3rd week of culture. For some TILs, FACS analysis was performed two or three times at different points. Figure 1b shows an example of TILs derived from case 1 that were determined at day 6 and day 14. All TILs cultured from PDXs stained positive for human CD45, suggesting that they originated from patients. Six of nine PDX-derived TILs stained predominantly positive for human CD3, demonstrating that the cells were mainly T lymphocytes. Further analysis found that five TILs were predominantly (> 70%) CD8^+^, and one was predominantly (70%) CD4^+^ (Fig. 1b, c).

**Fig. 2:**
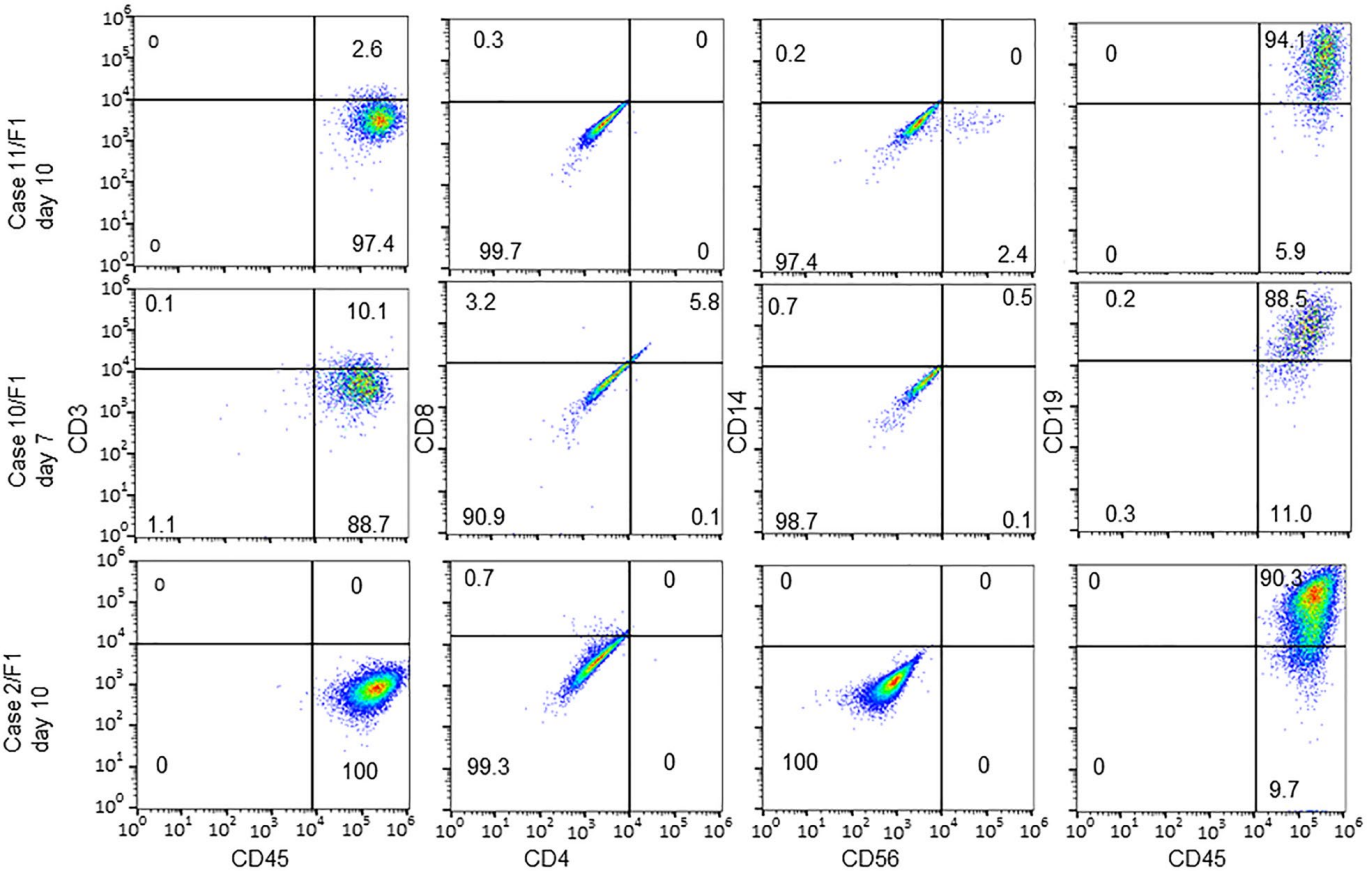
Flow cytometric profiles of three PDXs for which the cultured TILs were predominantly B cells. The numbers in each panel indicate the percentages of cells in each fraction. All antibodies used were mouse anti-human monoclonal antibodies

The FACS analysis found that TILs from three PDXs stained positive for human CD45 but negative for human CD3 (CD45^+^ CD3^−^). Further analysis indicated that these cells were negative for human CD8, CD4, CD14, and CD56 but positive for human CD19 (Fig. 2), suggesting these were B cells derived from the patients. Together, these results suggest that both T cells and B cells may co-exist within PDX.

### CD8^+^ T cells in PDXs were predominantly effector memory T cells

To further characterize the TILs derived from PDXs, we isolated DNA from TILs derived from two PDXs and performed DNA fingerprint analysis together with analysis of DNA from the primary tumors and/or peripheral blood mononuclear cells (PBMCs) from the same patients. The results showed that DNA from TILs matched with DNA from the primary tumor and PBMCs, demonstrating that the TILs were derived from the patients.

**Fig. 3:**
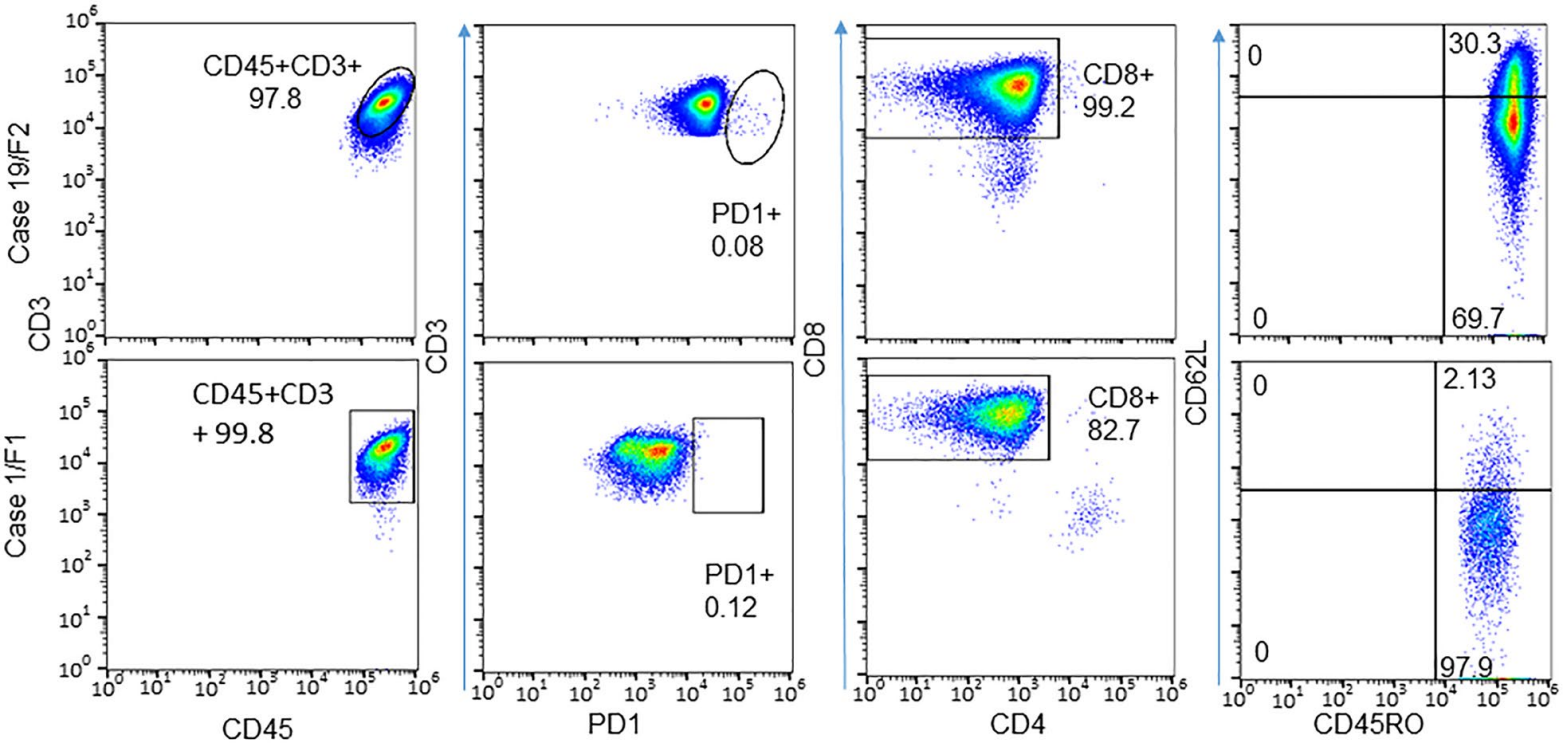
Flow cytometric profiles of CD8^+^ TILs from PDXs. Two CD8^+^ TILs from PDXs were analyzed for CD45RO and CD62L. The results showed that both were CD45RO+, with a portion of cells that were also CD62L+

**Fig. 4:**
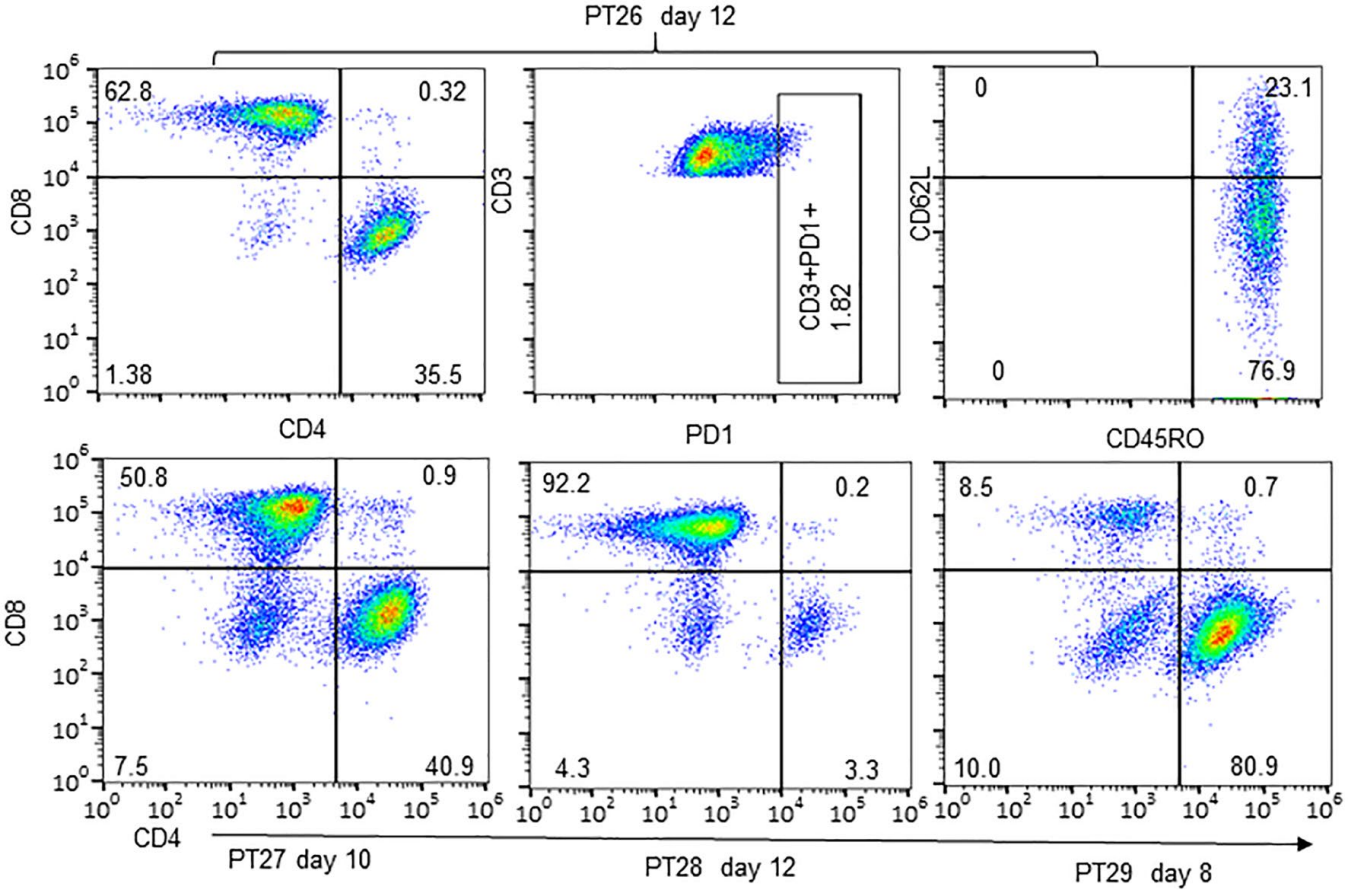
TILs cultured from primary NSLCL tumor specimens. Top panel: expressions of PD-1, CD45RO, and CD62L in TILs cultured from the primary tumor PT26. Bottom panel: percentage of CD4^+^ and CD8^+^ TILs cultured from primary tumors of three other NSCLC cases. The demographic information and clinical diagnoses for these four NSCLC cases were shown in Additional file 1: Table S2

To determine subtypes of CD8^+^ TILs in PDXs, we analyzed expressions of PD-1, CD45RO, and CD62L. FACS analysis for CD8^+^ TILs derived from two PDXs showed that these cells were negative for the exhaustion marker PD-1 but expressed a high level of CD45RO (Fig. 3), a marker for primed and memory cells [29, 30], suggesting that these CD8^+^ cells were likely memory cells. Expression of CD62L was low in about 70 to 98% of CD45RO-positive cells, indicating these cells were mainly effector memory T cells. This cell subtype pattern was similar to that of TILs cultured from a primary NSCLC tumor, which also showed low PD-1 expression, with high CD45RO expression and low CD62L expression (Fig. 4). It is not clear whether the low PD-1 expression was caused by in vitro culture with IL-2, as reported by others [31, 32]. However, we found that presence of CD4^+^ cells was more frequent in TILs cultured from primary tumors of NSCLC patients than in TILs cultured from PDXs, although the percentages of CD4^+^ cells varied among cases (Fig. 4).

### TILs exist in both stroma and intraepithelial cancer structures of PDXs

**Fig. 5:**
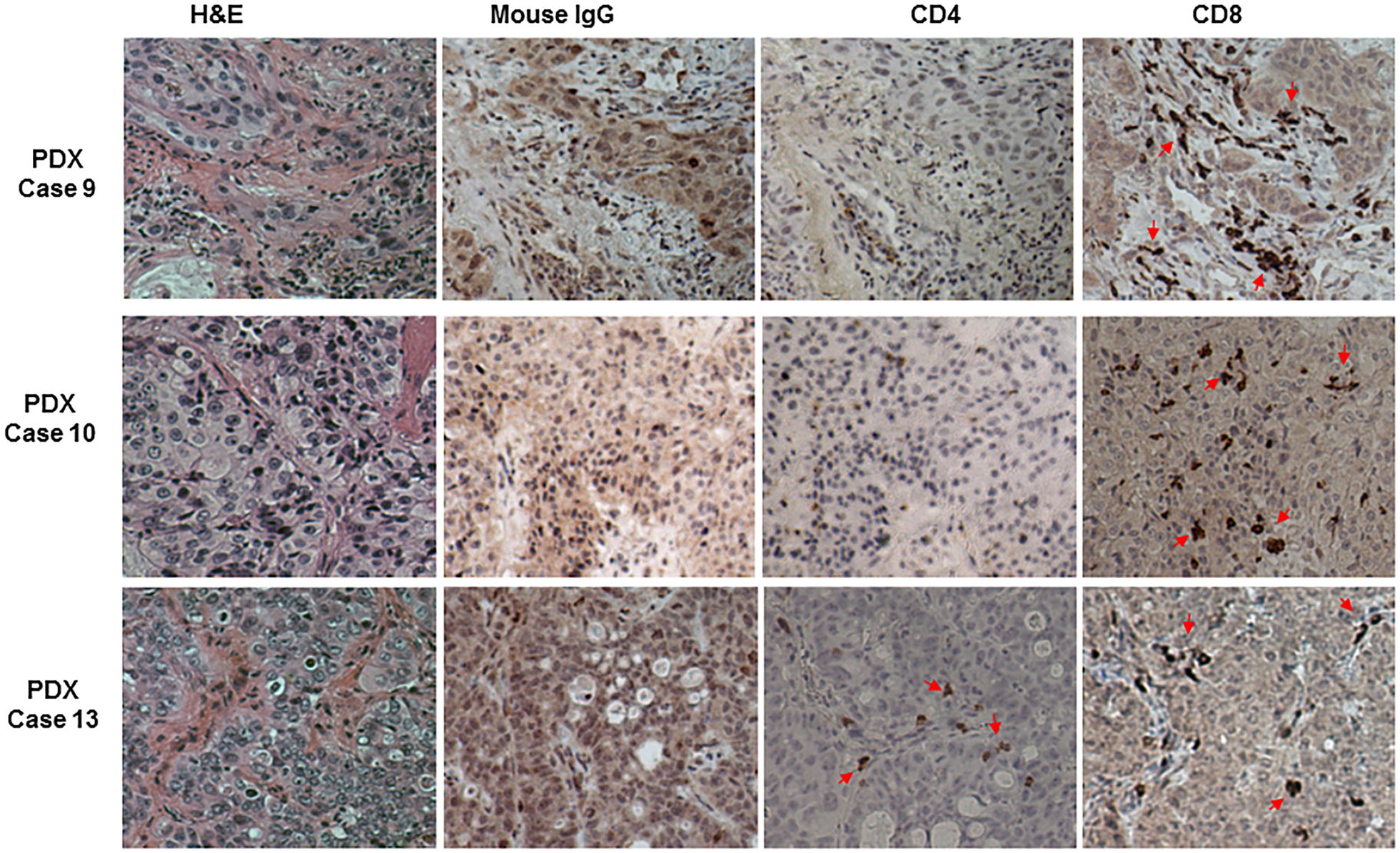
Immunohistochemical staining of PDXs. Examples of three PDXs derived from lung adenocarcinoma (cases 9 and 13) and squamous cell cancer (case 10) were stained with mouse anti-human CD4 and CD8 antibodies. CD8^+^ T cells were detected in all three samples, whereas CD4^+^ T cells were detected in case 13. Case 9 had CD8^+^ cells mainly in the stroma, case 10 had CD8^+^ cells mainly in the intraepithelial cancer structure, and case 13 had CD8^+^ cells in both stroma and intraepithelial cancer structure. Arrows indicate positive cells

To determine TIL distribution inside PDXs, we performed immunohistochemical analysis of 12 PDXs for which formalin-fixed paraffin-embedded PDX sections were available. The tissue sections were stained with mouse monoclonal antibodies specific for human CD4 or CD8. Of twelve cases analyzed, CD4^+^ T cells were detected in two PDXs while CD8^+^ T cells were detected in four PDXs. The TILs were detected in both stroma and intraepithelial cancer structures of PDXs (Fig. 5). However, we found some discrepancy between TILs detectable in cultures and in immunohistochemical staining. For example, we did not obtain TILs in culture but detected TILs in the tissue section for the case 9. In contrast, TILs were obtained in culture for cases 5 and 19 but were not detected in their tissue sections. This discrepancy may reflect spatial heterogeneity of TIL distribution within PDXs and/or the process of TIL enrichment during culture.

**Fig. 6:**
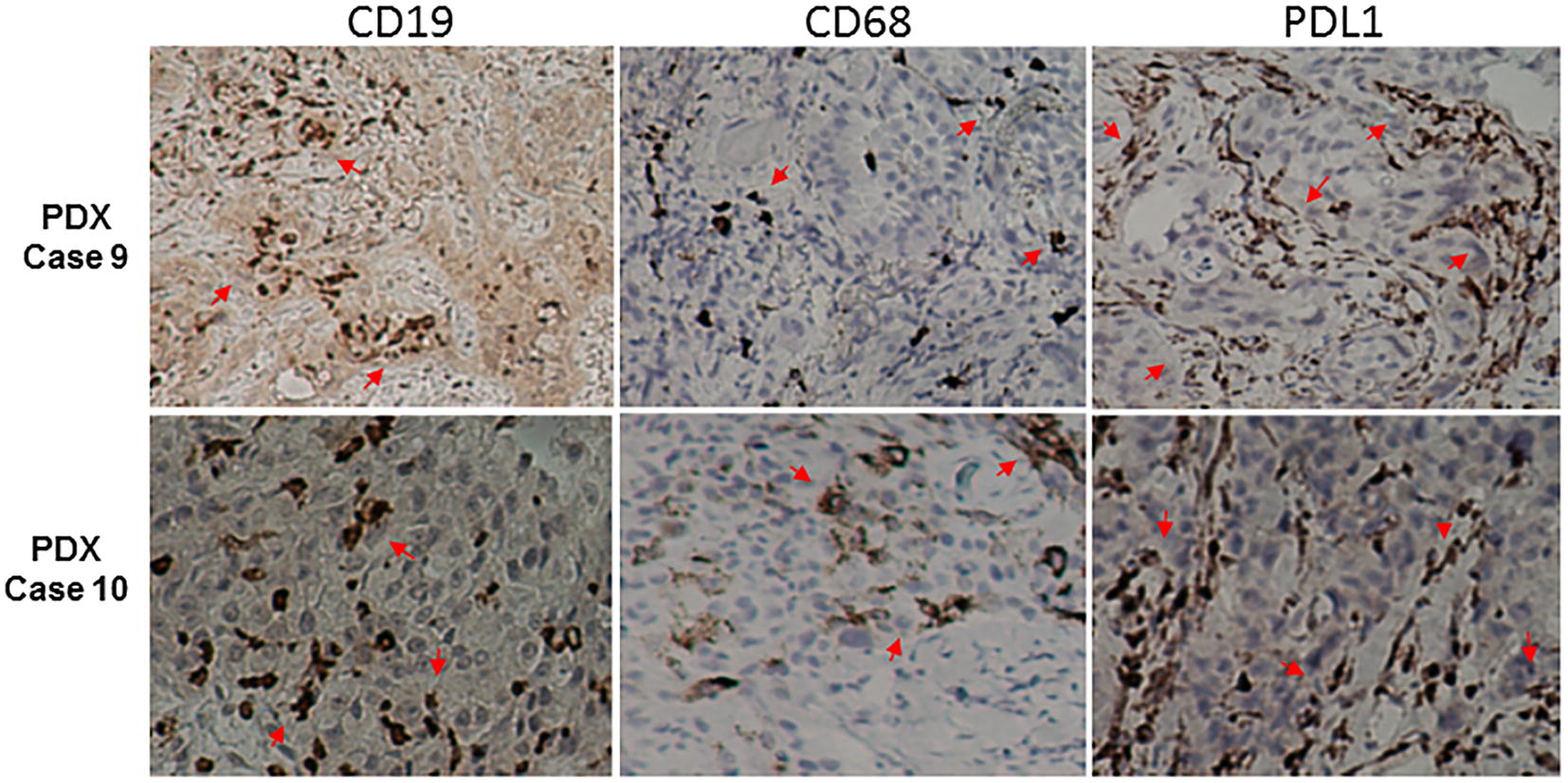
Immunohistochemical staining of two PDXs with anti-human CD19, CD68, and PD-L1 antibodies. Human CD19^+^ and CD68^+^ cells were detected in the stroma and/or the intraepithelial cancer structure, while PD-L1^+^ cells were mainly detected in the in stroma. Arrows indicate positive cells

We further tested two PDXs for the presence of human B cells and macrophages and expression of human PD-L1 by immunohistochemical staining. The results showed that human B cells and macrophages were present in these two PDXs, either in stroma or intraepithelial cancer structures (Fig. 6). Moreover, human PD-L1 was highly expressed in stromal cells of these two PDXs. Together, our results suggest that PDXs may preserve immune microenvironments of human cancers and might be used for evaluating the tumor immune microenvironment or anticancer immunotherapies.

### TILs were preserved in cryopreservation of PDXs, passaged to the F2 generation, and migrated to spleen of PDX-bearing mice

**Fig. 7:**
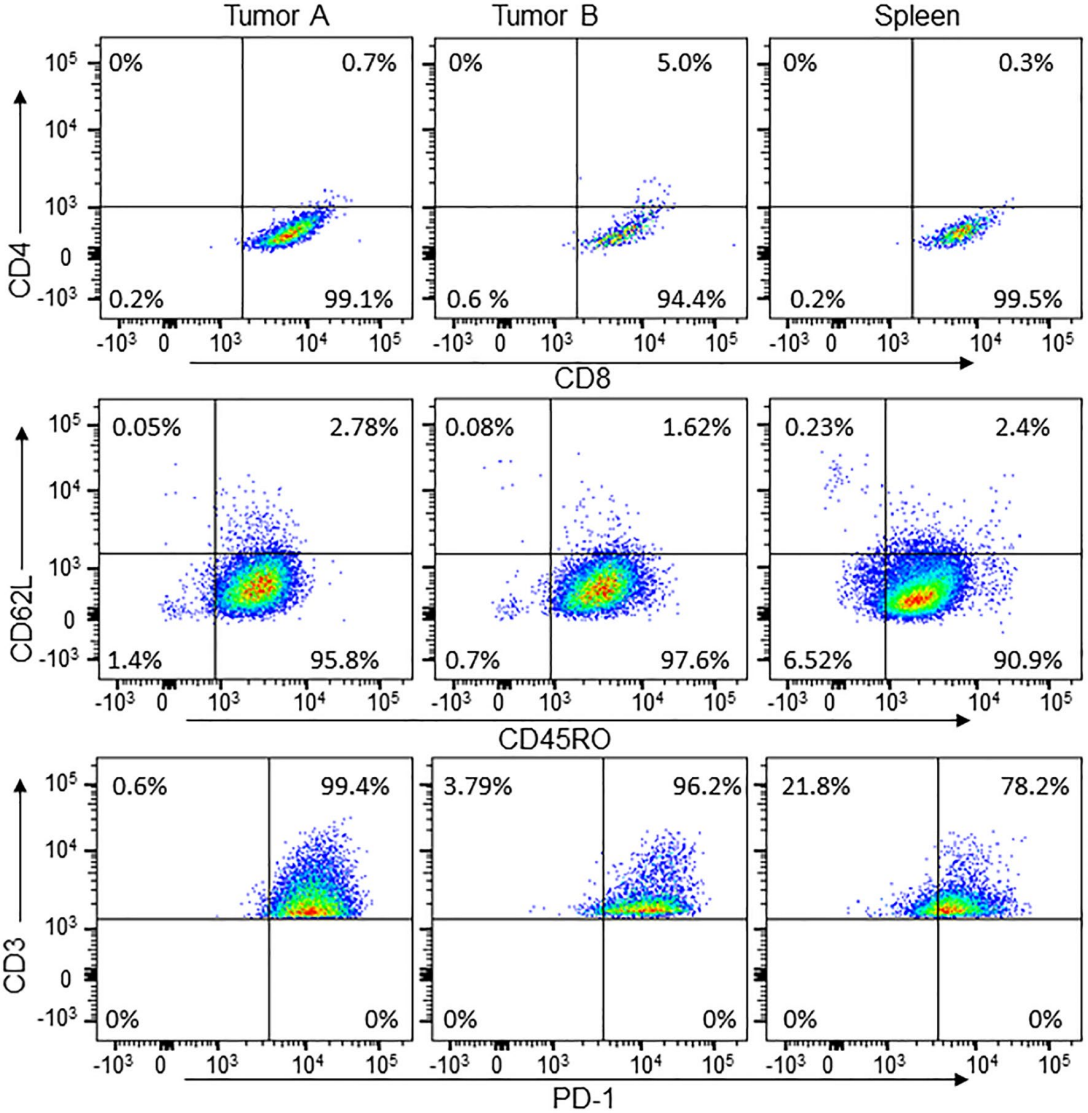
Flow cytometric analysis of F2 PDX of case 9. The F2 PDX was generated from cryopreserved F1 PDX tissue of case 9 in two NSG mice (Tumor A and B). The spleens of the PDX-bearing mice were also tested for human lymphocytes. The graphs showed that both tumors and spleens had human CD8^+^ lymphocytes, which were CD45RO^+^/CD62L^−^ and PD-1^+^

As TILs were cultured from the F2 generation of PDX (Table 1), we further tested whether patient-derived immune cells in PDX can be passaged to the F2 generation after cryopreservation of PDXs. To this end, we restored cryopreserved F1 PDX tissue from case 9, and a small piece of tumor tissue (about 2 mm in diameter) was re-implanted to two NSG mice to generate F2 PDXs. When F2 PDXs reached about 15 mm in diameter (58 days after implantation), we harvested PDXs and the spleens from the mice and tested these tissues for the presence of human T lymphocytes with anti-human CD3, CD4, CD8 antibodies. Flow cytometric analysis showed that human CD8^+^ T cells were detected in F2 PDXs harvested from both mice (Fig. 7, Tumor A and B). We also detected human T cells in the spleens of these PDX-bearing mice. The CD8^+^ cells in both tumors and in the spleens were CD45RO^+^/CD62L^−^ and PD-1^+^. This result demonstrated that patient-derived immune T cells can be preserved during cryopreservation of PDXs and passaged in early generation of PDXs. These T cells can also migrate to other organs, such as the spleen, of PDX-bearing mice.

## Discussion

Our results demonstrated that patient-derived T cells and/or B cells co-exist in some PDXs and that these immune cells may be passed in early passages of PDXs. We also found that the T cells in the PDXs were predominantly CD8^+^ effector memory cells, which were present in both stroma and intraepithelial cancer structures and could be passaged in early generations of PDXs. The functions of these immune cells remain to be further characterized.

Because PDXs are usually established in immunodefective mice—particularly in NSG mice, which are defective in T, B, and NK cells and have a weakened innate immune system [33]—PDXs are considered inappropriate for evaluation of immunotherapy unless human immune components are reconstituted in so-called humanized mice. Reconstitution of the human immune system in immunodeficient mice is feasible by transplantation of human CD34^+^ hematopoietic stem cells or human cord blood cells [34, 35]. The reconstituted immune cells can infiltrate into the human xenograft tumors transplanted in the humanized mice, providing immune microenvironments for biological characterization and therapeutic evaluation. Indeed, such humanized mice bearing PDX models were reported recently for evaluation of efficacy and mechanisms of PD-1-targeted cancer immunotherapy [36]. As immunotherapy emerges as the first- or second-line treatment for multiple cancer types, the application of xenograft human tumor models with immune microenvironments is expected to increase in future preclinical studies.

A possible limitation of humanized mouse models is the allogeneic graft-versus-tumor activity caused by human leukocyte antigen (HLA) mismatch if the immune cells and PDX are not derived from the same person, although humanized NSG mice have been reported to support growth of partially HLA-matched PDX tumors [36]. An alternative approach is to co-transplant immune cells and tumor tissue derived from the same patient. Nevertheless, infusion of human PBMCs in NSG mice is known to cause graft-versus-host disease [36]. Thus, other alternative models with tumor cells and immune cells derived from the same patients will be required for investigating the role of the immune microenvironment in cancer biology and in cancer immunotherapy. The co-existence of patient-derived immune cells in some PDXs and passaged in the F2 generation may provide such an alternative model for preclinical studies.

Our results showed that about 35% of lung PDXs tested have patient-derived immune cells in the first or second passages, and these immune cells survived for up to 290 days in vivo in mice. The patient-derived lymphocytes can enter the circulation and migrate to the spleen of PDX-bearing mice. This result showed that at least some early-passage PDXs might be used for preclinical studies that require an immune microenvironment. Because both tumor graft and immune cells were derived from the same patients, allogeneic immune response caused by HLA mismatch could be avoided. Nevertheless, this study also had limitations because, due to limited in vitro expansion of TILs, the functionality and clonal diversity of the PDX-derived TILs remain to be characterized. It is not yet clear whether these tumor-resident lymphocytes can recognize some tumor-associated antigens. It is also not clear whether these TILs are sufficient in numbers for in vivo activation or mobilization for evaluating cancer immunotherapy. The evidence of patient-derived immune cells co-existing in PDXs indicates that lung PDXs might be useful for evaluating the functionality of immune components in tumor microenvironments.

## Conclusions

Our results showed that patient-derived tumor immune microenvironments co-exist in early passages of PDXs for up to 290 days. The human immune cells detected in PDXs include CD8^+^ T cells, CD4^+^ T cells, B cells, and macrophages. These immune cells originated from the same patients as the PDXs and were present in stroma or intraepithelial cancer structures. High levels of human PD-L1 expression were also detected in PDXs. These results suggest that PDXs can be used for evaluating the functionality of immune components in tumor microenvironments.

## Additional file

**Additional file 1: Table S1.** Clinical information of PDXs used for TIL studies. **Table S2.** Clinical information of fresh tumor samples used for TIL culture and analysis.

## Abbreviations

HLA: human leukocyte antigen
IL-2: interleukin-2
NSCLC: non-small cell lung cancer
NSG: non-obese diabetic/severe combined immunodeficiency (NOD-SCID) mice with null mutations of the gene encoding for the IL-2 receptor-γ
PBMCs: peripheral blood mononuclear cells
PDX: patient-derived xenograft
TIL: tumor-infiltrating lymphocyte
