# Supplementary material for: Patient-derived tumor immune microenvironments in patient-derived xenografts of lung cancer
Source: J Transl Med. 2018 Nov 26;16:328. doi: 10.1186/s12967-018-1704-3 (PMC6260563; doi:10.1186/s12967-018-1704-3)
Supplement: Supplementary file 1 — Additional file 1: Table S1. Clinical information of PDXs used for TIL studies. Table S2. Clinical information of fresh tumor samples used for TIL culture and analysis. [file 12967_2018_1704_MOESM1_ESM.pdf]

**Additional file 1: Table S1 Clinical information of PDXs used for TIL studies**

| Case ID | Age | Sex | Ethnicity | Pathology | Grades | Stages | Tumor Volume | Smoke status | Pretreatment | Passage | In vivo Days |
|---|---|---|---|---|---|---|---|---|---|---|---|
| 1 | 70 | F | White | adenocarcinoma | Moderate | T2aN0M0 | 7.62 | Former | NO | F1 | 292 |
| 2 | 77 | M | White | adenocarcinoma | Moderate | T2bN1M0 | 156.00 | Former | Carboplatin/Pemetrexed | F1 | 90 |
| 3 | 65 | F | White | adenocarcinoma | Poor | T4N2M0 | 7.20 | Former | NO | F1 | 181 |
| 4 | 74 | M | White | adenocarcinoma | Moderate | T3N0M0 | 0.72 | Former | NO | F1 | 166 |
| 5 | 66 | F | White | adenocarcinoma | Poor | T2bN0M0 | 110.00 | Former | NO | F1 | 128 |
| 6 | 70 | F | White | squamous cell ca. | Moderate | T3N2M1 | 273.00 | Former | NO | F1 | 98 |
| 7 | 39 | F | White | adenocarcinoma | Moderate | T2aN1M0 | 29.64 | Never | NO | F1 | 92 |
| 8 | 72 | F | White | squamous cell ca. | Moderate | T2aN0M0 | 63.00 | Former | NO | F1 | 144 |
| 9 | 83 | M | White | adenocarcinoma | Moderate | T2aN0M0 | 45.15 | Former | NO | F1 | 88 |
| 10 | 62 | M | White | squamous cell ca. | Poor | T2bN2MO | 122.50 | Current | NO | F1 | 69 |
| 11 | 66 | F | White | adenocarcinoma | Poor | T2bN1M0 | 96.00 | Former | Cisplatin/Docetaxel | F1 | 64 |
| 12 | 70 | F | White | adenocarcinoma | Moderate | T2aN0M1 | 43.74 | Current | NO | F1 | 121 |
| 13 | 59 | F | White | adenocarcinoma | Poor | T3N1M0 | 21.00 | Former | NO | F1 | 98 |
| 14 | 82 | F | White | adenocarcinoma | Moderate | T3N0M0 | 162.00 | NA | NO | F2 | 275 |
| 15 | 68 | M | White | adenocarcinoma | Moderate | T1N0M0 | 1.50 | Former | NO | F2 | 514 |
| 16 | 70 | F | Hispanic | adenocarcinoma | Well | T3N0M0 | 157.50 | NA | NO | F2 | 97 |
| 17 | 65 | F | White | adenocarcinoma | Poor | T2aN1M1 | 25.20 | Former | NO | F2 | 315 |
| 18 | 54 | F | Black | giant/spindle cell | Poor | T3N2M0 | 48.00 | Former | NO | F2 | 252 |
| 19 | 55 | M | White | adenocarcinoma | Poor | T3N2M0 | 20.25 | Former | Cisplatin/Pemetrexed | F2 | 84 |
| 20 | 70 | F | White | squamous cell ca. | poor | T1bn0m0 | 9.35 | Current | NO | F2 | 211 |
| 21 | 67 | F | White | adenocarcinoma | NA | TxN2M1 | | Former | Carboplatin/Pemetrexed | F2 | 239 |
| 22 | 57 | F | Asian | neuroendocrine ca. | Well | T2aN0M0 | 27.00 | Former | NO | F2 | 237 |
| 23 | 85 | M | White | adenosquamous ca. | poor | T2bN1M0 | 73.30 | Former | NO | F2 | 136 |
| 24 | 80 | F | White | squamous cell ca. | poor | T3N1M1 | 110.00 | Former | NO | F2 | 125 |
| 25 | 70 | M | Hispanic | pleomorphic | Poor | T3N0M0 | 420.00 | Former | NO | F2 | 136 |

Additional file 1: Table S2 Clinical information of fresh tumor samples used for TIL culture and analysis

| Case ID | Age | Sex | Ethnicity | Pathology | Grades | Stages | Tumor Volume (cm3) | Smoke status | Pretreatment |
|---|---|---|---|---|---|---|---|---|---|
| PT26 | 78 | M | White | Squamous cell ca. | Moderate | T2N0M0 | 43.4 | Former | No |
| PT27 | 57 | M | White | Squamous cell ca. | Moderate | T1N0M0 | 19.2 | Never | Cisplatin/Taxol |
| PT28 | 41 | M | White | Neuroendocrine ca. | Moderate | T4N1MX | 169.2 | Never | Cisplatin/etoposide |
| PT29 | 66 | F | White | Adenocarcinoma | Well | T1N1M0 | 13.3 | Former | No |
